# Noncontrast Computed Tomography Signs as Predictors of Hematoma Expansion, Clinical Outcome, and Response to Tranexamic Acid in Acute Intracerebral Hemorrhage

**DOI:** 10.1161/STROKEAHA.119.026128

**Published:** 2019-11-18

**Authors:** Zhe Kang Law, Azlinawati Ali, Kailash Krishnan, Adam Bischoff, Jason P. Appleton, Polly Scutt, Lisa Woodhouse, Stefan Pszczolkowski, Lesley A. Cala, Robert A. Dineen, Timothy J. England, Serefnur Ozturk, Christine Roffe, Daniel Bereczki, Alfonso Ciccone, Hanne Christensen, Christian Ovesen, Philip M. Bath, Nikola Sprigg

**Affiliations:** 1 From the Stroke Trials Unit, Division of Clinical Neuroscience (Z.K.L., A.A., A.B., J.P.A., P.S., L.W., S.P., T.J.E., N.S., P.M.B), University of Nottingham, United Kingdom; 2 Radiological Sciences (S.P., R.A.D.), University of Nottingham, United Kingdom; 3 NIHR Nottingham Biomedical Research Centre (RD) and Vascular Medicine, Division of Medical Sciences and GEM (T.J.E.), University of Nottingham, United Kingdom; 4 Department of Medicine, National University of Malaysia, Kuala Lumpur, Malaysia (Z.K.L); 5 Department of Stroke, Nottingham University Hospitals NHS Trust, United Kingdom (K.K., N.S., P.M.B); 6 School of Medicine, University of Western Australia, Perth, Australia (L.A.C.); 7 Department of Neurology, Selcuk University Medical Faculty, Konya, Turkey (S.O.); 8 Institute for Applied Clinical Studies, Keele University, Staffordshire, Stoke-on-Trent, United Kingdom (C.R.); 9 Department of Neurology, Semmelweis University, Budapest, Hungary (D.B.); 10 Neurology Unit, Azienda Socio Sanitaria Territoriale di Mantova, Mantua, Italy (A.C.); 11 Department of Neurology, Bispebjerg and Frederiksberg Hospital, University of Copenhagen (C.O., H.C.); 12 Copenhagen Trial Unit, Centre for Clinical Intervention Research, Rigshospitalet, Copenhagen University Hospital, Denmark (C.O.).

**Keywords:** cerebral hemorrhage, hematoma, odds ratio, prevalence, probability, tranexamic acid

## Abstract

Blend, black hole, island signs, and hypodensities are reported to predict hematoma expansion in acute intracerebral hemorrhage. We explored the value of these noncontrast computed tomography signs in predicting hematoma expansion and functional outcome in our cohort of intracerebral hemorrhage.

**Methods—:**

The TICH-2 (Tranexamic acid for IntraCerebral Hemorrhage-2) was a prospective randomized controlled trial exploring the efficacy and safety of tranexamic acid in acute intracerebral hemorrhage. Baseline and 24-hour computed tomography scans of trial participants were analyzed. Hematoma expansion was defined as an increase in hematoma volume of >33% or >6 mL on 24-hour computed tomography. Poor functional outcome was defined as modified Rankin Scale of 4 to 6 at day 90. Multivariable logistic regression was performed to identify predictors of hematoma expansion and poor functional outcome.

**Results—:**

Of 2325 patients recruited, 2077 (89.3%) had valid baseline and 24-hour scans. Five hundred seventy patients (27.4%) had hematoma expansion while 1259 patients (54.6%) had poor functional outcome. The prevalence of noncontrast computed tomography signs was blend sign, 366 (16.1%); black hole sign, 414 (18.2%); island sign, 200 (8.8%); and hypodensities, 701 (30.2%). Blend sign (adjusted odds ratio [aOR] 1.53 [95% CI, 1.16–2.03]; *P*=0.003), black hole (aOR, 2.03 [1.34–3.08]; *P*=0.001), and hypodensities (aOR, 2.06 [1.48–2.89]; *P*<0.001) were independent predictors of hematoma expansion on multivariable analysis with adjustment for covariates. Black hole sign (aOR, 1.52 [1.10–2.11]; *P*=0.012), hypodensities (aOR, 1.37 [1.05–1.78]; *P*=0.019), and island sign (aOR, 2.59 [1.21–5.55]; *P*=0.014) were significant predictors of poor functional outcome. Tranexamic acid reduced the risk of hematoma expansion (aOR, 0.77 [0.63–0.94]; *P*=0.010), but there was no significant interaction between the presence of noncontrast computed tomography signs and benefit of tranexamic acid on hematoma expansion and functional outcome (*P* interaction all >0.05).

**Conclusions—:**

Blend sign, black hole sign, and hypodensities predict hematoma expansion while black hole sign, hypodensities, and island signs predict poor functional outcome. Noncontrast computed tomography signs did not predict a better response to tranexamic acid.

**Clinical Trial Registration—:**

URL: https://www.isrctn.com. Unique identifier: ISRCTN93732214.

## Abstract 2

Supplemental Digital Content is available in the text.

Hematoma expansion complicates up to 38% of patients with acute intracerebral hemorrhage (ICH) within the first few hours of onset and leads to higher mortality and morbidity.^1^ However, given that the majority of patients with ICH do not have hematoma expansion, identifying patients at risk of hematoma expansion may be important in clinical trials testing hemostatic therapies to selectively target patients who are most likely to benefit.

Shorter onset-to-computed tomography (CT) time, larger baseline hematoma volume, prior antiplatelet and anticoagulant therapy were identified as independent predictors of hematoma expansion with *C*-index of 0.78 (95% CI, 0.75–0.82) in a large patient level meta-analysis of data from cohort studies and randomized trials.^2^ The predicted probability of hematoma expansion increased with larger bleeds and peaked at ≈75 mL before declining while the probability of hematoma expansion was the highest in patients with onset-to-CT time of <3 hours.^2^ CT angiography (CTA) spot sign was reported to have a sensitivity of ≈60% and specificity of 90% in predicting hematoma expansion but the addition of spot sign only increased the *C*-index by 0.05.^2,3^ Furthermore, CTA is not routinely performed in patients with ICH. In the TICH-2 trial (Tranexamic acid for IntraCerebral Hemorrhage-2), only 10% of patients had a baseline CTA.^4^

Several noncontrast CT (NCCT) signs of heterogeneous density and irregular shape have been identified as predictors of hematoma expansion in ICH. The blend sign, black hole sign, swirl sign, fluid level, and hypodensities are signs of heterogeneous density; another, the island sign, reflects irregular shape.^5–10^ Heterogeneous density represents areas of hyperdense mature blood and hypodense fresh blood indicating ongoing bleeding,^10^ while island sign may represent multifocal bleeding points.^7^ The universal availability of NCCT in patients with ICH and the reportedly excellent interrater reliability amongst trained assessors (κ, 0.806–0.957) makes these signs an attractive alternative to CTA spot sign.^5–8^ These signs have a sensitivity of 31.9% to 44.7% and specificity of 94.7% to 98.2% for prediction of hematoma expansion.^5–8^ However, many source studies were small single-center studies.^5–8^ One exception was the Antihypertensive Treatment of Acute Cerebral Hemorrhage II, which found NCCT signs to be useful predictors of hematoma expansion.^11^ Several meta-analyses have found substantial heterogeneity between the studies.^12,13^ Therefore, further studies are needed to evaluate the value of NCCT signs.

In the TICH-2 trial, there was no significant difference in shift of functional outcome between the tranexamic acid and placebo group despite a significant reduction in risk of hematoma expansion in the tranexamic acid group.^4^ In the current analysis, we explore the role of some of the NCCT signs as predictors of hematoma expansion and poor functional outcome, and if a subgroup of patients with 1 or more of these signs benefited from tranexamic acid.

## Methods

The TICH-2 trial was a prospective randomized placebo controlled trial testing the efficacy and safety of intravenous tranexamic acid in patients with acute spontaneous ICH presenting within 8 hours of symptom onset. Details of the trial were previously published.^4,14^ Ethics approval was obtained from the local institutional review board. Written informed consent was obtained from patients or relatives before enrolment. After publication of the planned primary and secondary analyses, the trial data can be shared on reasonable request to the corresponding author and trial steering committee.

### Image Acquisition

An NCCT was required for the diagnosis of spontaneous ICH before recruitment and randomization. All CT brain scans were performed as per local protocol. CT scans obtained from any scanner of any manufacturer; any slice thickness with a minimum of an axial view was accepted. CT scans with incomplete or missing slices were excluded. Baseline scans were performed before randomization. Follow-up CT scans were performed at 24±12 hours after the baseline scans.^14^ When multiple scans were available, the scan closest to 24 hours after randomization was used to determine hematoma expansion. The exception was if the 24-hour CT was performed after neurosurgery, when a preoperative CT scan was used instead. A participant would be excluded from analysis for hematoma expansion if no follow-up scans or only postsurgery scans were available.

### Image Analysis

Three independent raters blinded to clinical data (Z.K. Law, neurologist; A. Ali, CT radiographer; K. Krishnan, stroke physician) performed volumetric measurements using the ITK-SNAP software version 3.6.0.^15^ Intraparenchymal and intraventricular hematoma volumes were computed independently using an active contour semiautomated segmentation algorithm,^15^ followed by manual editing if necessary.

Evaluation of intra- and interrater reliability of hematoma volume measurement was performed. Each rater performed 2 measurements on the same CT scan at least 1 day apart. In addition, to assess intraclass correlation, measurements were performed on 50 CT scans by all raters blinded to each other’s measurements.

**Figure 1.**
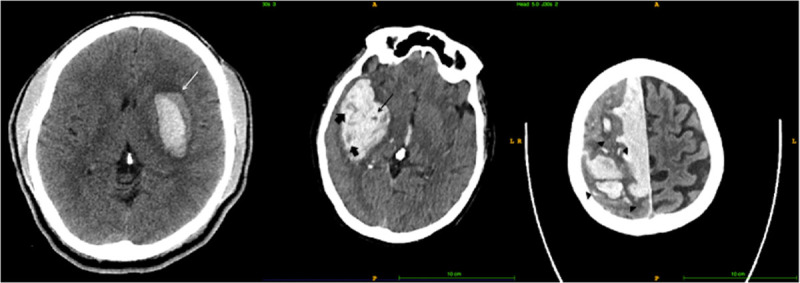
Shows the blend sign (left, white arrow), black hole sign (middle, black arrow), hypodensities (thick arrows), and island sign (right, black arrow heads showing multiple small islands).

Blend sign is defined as an area of hypodensity adjacent to the hyperdense area of hematoma. The margin should be well-defined with a difference of at least 18 Hounsfield units between the 2 areas (Figure 1).^5^ Black hole sign is an area of hypodensity that is completely encapsulated by an adjacent hyperdense area within a hematoma. The difference between the 2 areas should be at least 28 Hounsfield units.^6^ Black hole sign is a subset of hypodensities, which can be of any Hounsfield unit and have distinct or indistinct border, as long as it is completed encapsulated by hyperdense area.^8^ The island sign is characterized by presence of ≥3 separate small hematomas adjacent to the main hematoma or ≥4 small bubble- or sprout-like hematomas that are partially connected to the main hematoma.^7^ The signs were assessed by 4 independent raters (Z.K. Law, A. Ali, K. Krishnan, and A. Bischoff, medical student). All raters were trained and interrater reliability assessed using 70 scans, with raters blinded to each other’s ratings of signs.

### Outcomes and Statistical Analysis

For this analysis, the outcomes were based on the TICH-2 CTA statistical analysis plan^16^ as below.

#### Radiological Outcomes

Hematoma expansion is defined as increase in intraparenchymal hematoma volume on follow-up scan of >33% or > 6 mL from baseline hematoma. Total ICH expansion, defined as expansion of combined intraparenchymal and intraventricular hematoma volume of >33% or > 6 mL were analyzed as an outcome as well, as hematoma may have extended into intraventricular hemorrhage with a preserved intraparenchymal hematoma volume.

In addition, we explored a composite outcome of hematoma progression, which was defined as intraparenchymal hematoma expansion or delayed intraventricular or subarachnoid extension or an absolute intraventricular hematoma expansion of ≥2 mL. When follow-up scans were not available, early neurological deterioration (≥4 points increase in National Institutes of Health Stroke Scale or drop in Glasgow Coma Scale score of ≥ 2) or death before day-2 clinical assessment was considered hematoma progression. The rationale of including neurological deterioration or death is to avoid excluding patients who were unfit to have a follow-up scan.

#### Clinical Outcome

Unfavourable functional outcome, defined as a dichotomized modified Rankin Scale of 4 to 6, was the clinical outcome of interest.

#### Statistical Analysis

The sensitivity, specificity, positive and negative predictive values of each NCCT sign were analyzed individually. Fleiss kappa was used to measure interrater reliability as there were >2 independent raters. Descriptive analyses used Student *t* test, Mann-Whitney *U* test, and χ^2^ tests as appropriate. Multivariable logistic regression analyses were used to identify predictors of hematoma expansion and unfavourable functional outcome. The multivariable model included minimization factors as a priori variables and variables that were significant confounders on univariate analysis (resulting in change of odds ratio ≥0.1 when included). We also performed sensitivity analyses using hematoma progression and total ICH expansion as outcomes. Ordinal logistic regression was performed as a sensitivity analysis to explore if presence of NCCT signs leads to a shift in modified Rankin Scale. To explore the diagnostic yield of NCCT signs in addition to known predictors, we performed receiver operating characteristics analysis for known predictors of hematoma expansion (prior antiplatelet, baseline hematoma volume, and onset-to-CT time) and known predictors plus NCCT signs.

In addition, to explore the effect of tranexamic acid in patients with NCCT signs, a logistic regression model was constructed stratified by status of NCCT signs. Ninety-five percent CIs are given and *P* of <0.05 were considered statistically significant. All analyses were performed using SPSS version 24 (IBM, Armonk, NY).

## Results

Two thousand three hundred twenty-five participants were recruited into the trial from 124 centers in 12 countries. Baseline scans were available in 2273 patients (97.8%). Of these, 2077 (89.3%) participants also had follow-up CT scans and were included in the analysis of hematoma expansion (Figure I in the online-only Data Supplement). In addition, 236 participants without follow-up scans had available neurological deterioration or death status at 48 hours, and 2313 patients (99.5%) were included in analysis of hematoma progression. Day 90 modified Rankin Scale was available in 2307 patients (99.2%).

Five hundred seventy participants (27.4%) had hematoma expansion while 1259 patients (54.6%) had poor functional outcome. Participants with hematoma expansion had higher National Institutes of Health Stroke Scale, lower Glasgow Coma Scale, larger baseline hematoma volumes, more lobar location, more likely to have had antiplatelet therapy before ICH, a shorter onset-to-CT time, and less intraventricular hemorrhage (Table 1). Participants with hematoma expansion were more likely to have blend sign, black hole sign, island sign, and hypodensities on baseline imaging (Table 1).

**Table 1.**
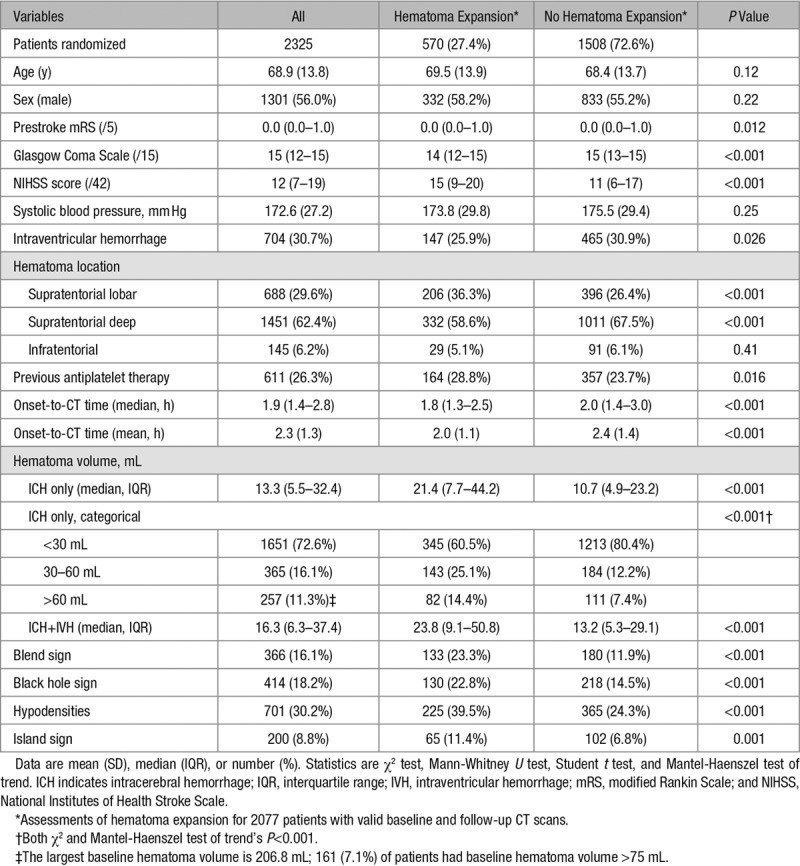
Baseline Characteristics in Patients With and Without Hematoma Expansion

The intraclass correlation for intraparenchymal hematoma volume was 0.94 (95% CI, 0.86–0.97). Intrarater Cohen κ for blend sign was 0.93 (95% CI, 0.79–1.00); black hole was 0.73 (95% CI, 0.45–1.00); island sign 0.85 (95% CI, 0.57–1.00); and hypodensities 0.66 (95% CI, 0.43–0.90). The interrater Fleiss κ for blend signs was 0.60 (95% CI, 0.47–0.74); black hole sign 0.53 (95% CI, 0.41–0.64); island sign 0.64 (95% CI, 0.53–0.75); and hypodensities 0.63 (95% CI, 0.39–0.86).

The prevalence of NCCT signs was: blend sign, 366 (16.1%); black hole sign, 414 (18.2%); island sign, 200 (8.8%); and hypodensities, 701 (30.2%). NCCT signs had low sensitivity (11.4%–39.5%) and high specificity (76%–93%) for hematoma expansion with island sign having the highest specificity but lowest sensitivity (Table I in the online-only Data Supplement). Similarly, NCCT signs had low sensitivity (14%–39%) and high specificity (79.5%–97.6%) for poor functional outcome (Table I in the online-only Data Supplement). Baseline hematoma volumes were significantly larger in all NCCT signs; patients with black hole sign and hypodensities had shorter onset-to-CT time while a larger proportion of patients with island signs were taking antiplatelet therapy (Table II in the online-only Data Supplement).

Univariate logistic regression showed that higher premorbid modified Rankin Scale, prior antiplatelet therapy, lower Glasgow Coma Scale, lobar location, larger baseline hematoma volume, shorter onset-to-CT time as well as the presence of blend sign, black hole sign, island sign, and hypodensities increased the odds of hematoma expansion (Table 2). As there was significant overlap between black hole sign and hypodensities, 2 different multivariable logistic regression models were constructed to predict hematoma expansion with model 1 including black hole sign and model 2 including hypodensities (Table 2). Baseline hematoma volume, onset-to-CT time, and prior antiplatelet therapy were significant predictors of hematoma expansion. Of the NCCT signs, blend sign, black hole, and hypodensities were significant independent predictors of hematoma expansion (Table 2). The island sign was no longer a significant predictor after adjusting for baseline hematoma volume. A combination of 1 or more NCCT signs did not improve the predictive probability of hematoma expansion (adjusted odds ratio [aOR], 1.57 [95% CI, 1.24–1.98]; *P*<0.001; not shown in table).

**Table 2.**
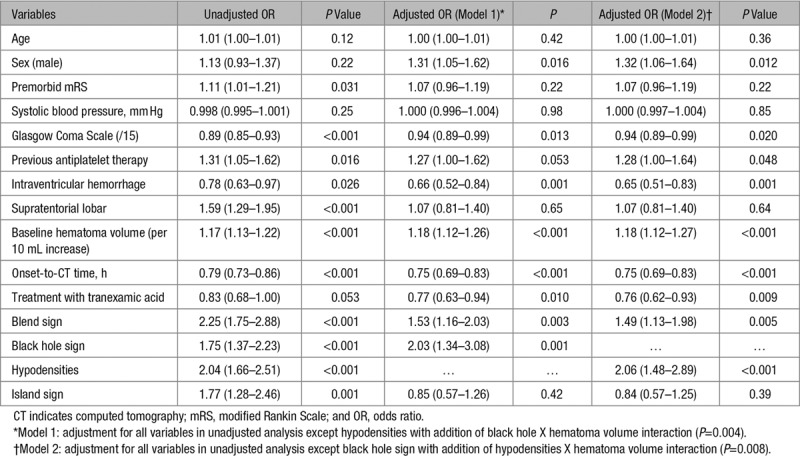
Binary Logistic Regression Analyses for Predictors of Hematoma Expansion

In the receiver operating characteristic analysis for hematoma expansion, the area under the curve for 3 predictors (hematoma volume, onset-to-CT time, and prior antiplatelet agent) was 0.654. Addition of blend and black hole signs, and blend sign and hypodensities improved the area under the curve minimally to 0.664 and 0.667, respectively.

As sensitivity analysis, we analyzed hematoma progression and total ICH volume expansion as outcomes. Multivariable logistic regression adjusting for variables used in the analysis for hematoma expansion showed that blend sign, black hole sign, and hypodensities were significant predictors of hematoma progression and total ICH expansion (Table III in the online-only Data Supplement).

Black hole sign, hypodensities, and island sign were significant predictors of poor functional outcome after adjustment of prognostic covariates (Table 3). A combination of 1 or more NCCT signs did not improve the predictive probability of poor functional outcome (aOR, 1.30 [1.01–1.66]; *P*=0.039; not shown in table). In the ordinal regression model, only the presence of black hole sign (aOR, 1.25 [1.01–1.56]; *P*=0.045) and hypodensities (aOR, 1.33 [1.10–1.61]; *P*=0.003) resulted in significant shift toward worse modified Rankin Scale (goodness-of-fit test, *P*=0.92; proportional odds assumption not violated).

**Table 3.**
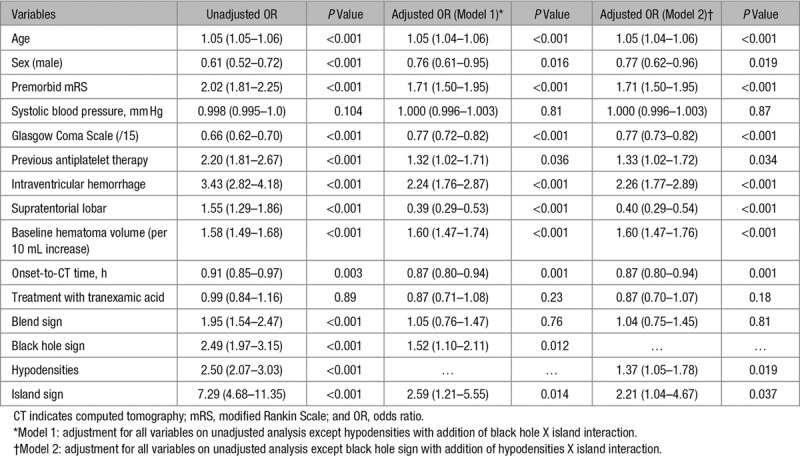
Binary Logistic Regression Analyses for Predictors of Death and Dependency (Dichotomized mRS Score of ≥4)

**Figure 2.**
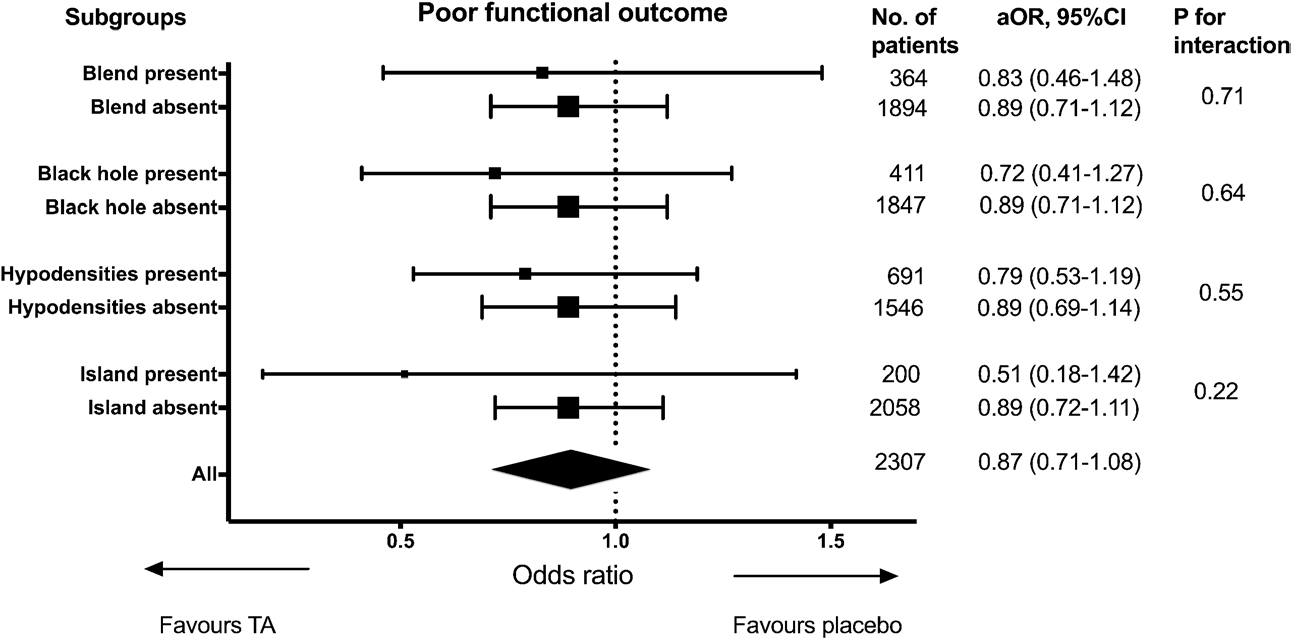
Effect of tranexamic acid on hematoma expansion, stratified by presence of noncontrast computed tomography signs. aOR indicates adjusted odds ratio.

**Figure 3.**
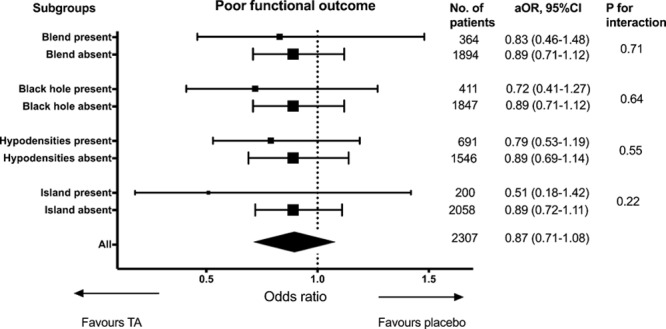
Effect of tranexamic acid on functional outcome, stratified by presence of noncontrast computed tomography signs. aOR indicates adjusted odds ratio.

Overall, tranexamic acid reduced the risk of hematoma expansion (aOR, 0.77 [0.63–0.94]; *P*=0.01). Similarly, there was significant reduction in hematoma progression (aOR, 0.71 [0.59–0.86]; *P*<0.001) and total ICH expansion (aOR, 0.82 [0.67–0.99]; *P*=0.047). Logistic regression model stratified by presence of NCCT signs did not show any significant interactions between the presence of signs and the benefit of tranexamic acid in reducing hematoma expansion (Figure 2). The effect of tranexamic acid on functional outcome, stratified by presence of NCCT signs is shown in Figure 3. Tranexamic acid did not significantly improve functional outcome regardless of the status of NCCT signs.

## Discussion

In the largest randomized controlled trial of hemostatic therapy in ICH, we found that blend sign, black hole sign, and hypodensities were independent predictors of hematoma expansion. Nevertheless, addition of these signs to more established predictors of onset-to-CT time, baseline hematoma volume, and antiplatelet therapy only marginally improved the area under the curve. The island sign was not a significant independent predictor of hematoma expansion.

Apart from Li et al who first described the blend and black hole signs, 2 large studies, Boulouis et al^8^ (n=1029) and Morotti et al^11^ (n=989) found them to be independent predictors of hematoma expansion. A recent meta-analysis of 5 studies (2248 patients) found the pooled sensitivity and specificity of blend sign to be 0.28 (0.16–0.46) and 0.92 (0.88–0.95), respectively.^17^ A second meta-analysis of 5 studies (1495 patients) reported a pooled sensitivity and specificity of 0.30 (0.20–0.41) and 0.91 (0.87–0.94), respectively for black hole sign.^18^ Our study similarly found blend and black hole signs to be highly specific but not sensitive for prediction of hematoma expansion.

Hypodensities had been found to be a useful predictor of hematoma expansion and poor functional outcome as well.^8,11^ There is significant overlap between black hole sign and hypodensities where black hole sign is a subset of hypodensities. We found hypodensities to be more sensitive but less specific than black hole. The adjusted odd ratio in predicting hematoma expansion of hypodensities was similar to that for the black hole sign (aOR, 2.06 versus 2.01).

On the contrary, we did not find the island sign to be an independent predictor of hematoma expansion. The island sign was of particular interest as it was reported to be very specific, albeit less sensitive, than the other NCCT signs. The island sign was first reported as a predictor of hematoma expansion and poor functional outcome in a single-center study by Li et al.^7^ Similarly, we found the island sign to have high specificity but low sensitivity in detecting hematoma expansion. However, after accounting for hematoma volume, the island sign did not independently predict hematoma expansion. This may be because island sign was mostly present in large hematomas (64.6 mL in island sign positive versus 11.8 mL in island sign negative); hence, hematoma volume was a significant confounder. Comparatively, the median volume was 30 mL in island sign positive hematoma in study by Li et al.^7^ Another reason is the prevalence of island sign in our cohort was relatively low (8.8% compared with 16% in study by Li et al) and the numbers may be inadequately powered to detect a difference.

Black hole sign, hypodensities, and island sign independently predicted poor functional outcome, although island sign did not predict hematoma expansion. Conversely, the blend sign that independently predicted hematoma expansion did not predict poor functional outcome. This suggests that although hematoma expansion increased the risk of poor functional outcome, there were other factors that contributed to poor functional outcome. This finding is in keeping with the trial main results where tranexamic acid significantly reduced hematoma expansion, early death (<7 days), and serious adverse events but did not improve functional outcome at day 90.^4^ We hypothesize that one of the mechanisms may be the development of perihematomal edema. Hematomas with island sign have a larger surface area of contact with the surrounding tissue and this may contribute to more inflammation and cerebral edema.

While some of the NCCT signs are useful predictors of hematoma expansion, none of the signs reliably predicted clinical benefit of tranexamic acid. This again supports the hypothesis of alternate pathological process contributing to poor outcome. Although tranexamic acid reduced the risk of hematoma expansion, the effect of tranexamic acid is short lasting with a half-life of 3 hours.^19^ It is therefore unlikely that tranexamic acid will have any biological effect on events occurring later such as perihematomal edema. In addition, the number of patients with NCCT signs is relatively small and may be inadequately powered to detect a modest benefit of tranexamic acid. The usefulness of the CTA spot sign in predicting clinical benefit of tranexamic acid is being studied in a separate substudy.^16^

Our findings of male sex, lower Glasgow Coma Scale, absence of IVH, prior antiplatelet therapy, larger baseline hematoma volume, and shorter onset-to-CT time as independent predictors of hematoma expansion were consistent with previous studies.^2^ Notably, although intensive blood pressure lowering can reduce hematoma expansion,^20^ systolic blood pressure on admission per se was not a predictor of hematoma expansion, a finding replicated in our study.^2^

The strength of the study is the large sample size and good availability of CT scans, making this the largest imaging analysis in ICH. There is a wide range of hematoma volumes from very small to very large hematoma reflective of real-world patients with ICH. To account for intraventricular extension and missing data, we performed exploratory analysis using total ICH expansion and a composite outcome of hematoma progression (data available in 99.5%) as outcomes. The analyses yielded similar results hence the conclusion is robust.

One limitation of this study is the interrater agreement is only moderate (κ, 0.53–0.64). The raters were from a range of background (physicians, radiographer, and medical student). Although all raters were trained before commencing assessment, the interpretation of the NCCT signs may not be as accurate as other studies where the assessments were usually performed by only 2 expert raters.^5–7,11^ Having more raters also reduced agreement as it is less likely for 4 raters to completely agree on the presence of signs compared with 2 raters. However, our interpretation represents a real world scenario where the raters are likely to have different background and experience.

Recently, there is much enthusiasm in exploring the role of NCCT signs in predicting hematoma expansion. We found previously established factors of baseline hematoma volume and onset-to-CT time to be more significant predictors of hematoma expansion. Addition of NCCT signs only improved the area under the curve minimally by 0.01 to 0.013. Hematoma volume may be the most important CT marker of hematoma expansion and may be used to better select in future hemostatic trials. Although we have used semiautomated segmentation technique for the volume measurement, simple clinical tool such as the ABC/2 method has been shown previously to be a reliable and feasible method in the context of clinical trials.^21^ In view of its prognostic value, calculation of hematoma volume should perhaps be recommended as a routine clinical practice.

## Conclusions

Blend sign, black hole sign, and hypodensities predict hematoma expansion while black hole sign, hypodensities, and island sign predict poor functional outcome. NCCT signs did not predict a better response to tranexamic acid.

## Acknowledgments

N. Sprigg and R. Dineen helped with study concept and design. Z.K. Law, A. Ali, and K. Krishnan measured hematoma volume and assessed for the presence of blend, black hole and island signs. A. Bischoff helped with assessment of black hole and island signs. R. Dineen, L. Cala, Alessandro Adami, and Ana Casado are neuroradiologists who performed adjudications. Z.K. Law performed the statistical analysis and wrote the first draft of the manuscript. All authors revised and approved the final draft.

## Sources of Funding

This article was supported by National Institute of Health Research Health Technology Assessment Programme (11_129_109) and Swiss Heart Foundation.

## Disclosures

N. Sprigg reports grants from National Institute for Health Research HTA and RfPB during the conduct of the study. P.M. Bath reports grants from National Institute for Health Research HTA and Research Councils United Kingdom during the conduct of the study; personal fees from Nestle, Sanofi, Diamedica, Moleac, and Phagenesis; nonfinancial support from Platelet Solutions. C. Roffe reports grants from National Institute for Health Research HTA and Research Councils United Kingdom during the conduct of the study; personal fees from Daiichi Sankyo, Allergan, Air Liquide, Merz, Boehringer, Bayer, Johnson and Johnson, Sanofi, Emtensor; nonfinancial support from European Stroke Conference and TRIDENT study team; other support from Firstkind Medical, Medtronic and Brainomix outside the submitted work. A. Ali received funding from National Institutes of Health. S. Pszczolkowski received funding from the British Heart Foundation. C. Ovesen received nonfinancial support from Merck Sharp and Dohme outside the submitted work.

## Supplementary Material
