# Supplementary material for: Noncontrast Computed Tomography Signs as Predictors of Hematoma Expansion, Clinical Outcome, and Response to Tranexamic Acid in Acute Intracerebral Hemorrhage
Source: Stroke. 2019 Nov 18;51(1):121–8. doi: 10.1161/STROKEAHA.119.026128 (PMC6924948; doi:10.1161/STROKEAHA.119.026128)
Supplement: Supplementary file 1 [file str-51-121-s001.pdf]

SUPPLEMENTAL MATERIALS

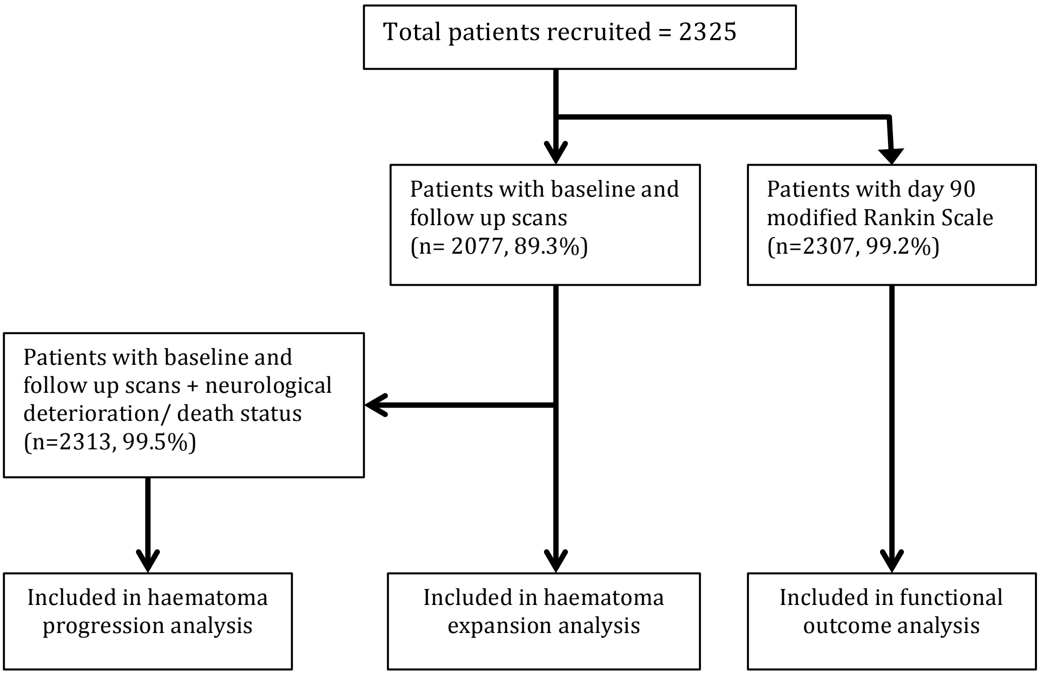

Figure I Flow chart showing numbers included in analysis.

Table I Diagnostic accuracy of NCCT signs for haematoma expansion and poor functional outcome

**Haematoma expansion**	Se	Sp	PPV	NPV	PLR	NLR	Accuracy
Blend sign	23.3% (19.9-27.0)	88.1% (86.3-89.7)	42.5% (37.6-47.5)	75.2% (74.3-76.1)	1.95 (1.60-2.39)	0.87 (0.83-0.91)	70.3% (68.3-72.3)
Black hole sign	22.8% (19.4-26.5)	85.5% (83.7-87.3)	37.4% (32.9-42.0)	74.6% (73.6-75.5)	1.58 (1.30-1.92)	0.90 (0.86-0.95)	68.3% (66.3-70.3)
Hypodensity	39.5% (35.5-43.7)	75.8% (73.5-77.9)	38.1% (35.0-41.4)	76.8% (75.5-78.1)	1.63 (1.42-1.87)	0.80 (0.74-0.86)	65.8% (63.7-67.9)
Island sign	11.4% (8.9-14.3)	93.2% (91.9-94.5)	38.9% (32.2-46.1)	73.6% (72.9-74.2)	1.69 (1.25-2.27)	0.95 (0.92-0.98)	70.8% (68.8-72.7)
Any NCCT sign	52.3% (48.1-56.5)	66.8% (64.3-69.2)	37.3% (34.9-39.8)	78.7% (77.1-80.3)	1.57 (1.42-1.75)	0.71 (0.65-0.78)	62.8% (60.7-64.9)
**Poor functional outcome (modified Rankin Scale of 4 to 6)**							
Blend sign	20.1% (17.9-22.4)	88.7% (86.6-90.6)	68.1% (63.5-72.4)	47.9% (47.1-48.8)	1.77 (1.45-2.18)	0.90 (0.87-0.93)	51.2% (49.1-53.3)
Black hole sign	24.0% (21.6-26.5)	88.8% (86.7-90.6)	72.0% (67.8-75.9)	49.2% (48.3-50.2)	2.14 (1.75-2.61)	0.86 (0.82-0.89)	53.4% (51.3-55.4)
Hypodensity	39.2% (36.5-42.0)	79.5% (76.9-81.9)	69.8% (66.8-72.6)	52.0% (50.7-53.4)	1.91 (1.66-2.20)	0.76 (0.72-0.81)	57.5% (55.4-59.5)
Island sign	14.3% (12.4-16.4)	97.8% (96.7-98.6)	88.5% (83.4-92.2)	48.6% (48.0-49.3)	6.39 (4.17-9.78)	0.88 (0.85-0.90)	52.2% (50.1-54.3)
Any NCCT sign	52.2% (49.3-55.0)	69.5% (66.6-72.3)	67.4% (65.0-69.7)	54.6% (52.9-56.4)	1.71 (1.54-1.90)	0.69 (0.64-0.74)	60.0% (58.0-62.1)

Se=sensitivity; Sp=specificity; PPV=positive predictive value; NPV=negative predictive value; PLR=positive likelihood ratio; NLR=negative likelihood ratio.

Table II Comparison of baseline haematoma volume, onset to CT time and history of antiplatelet between patients with and without noncontrast CT signs

	Baseline HV (mL)	Onset to CT time (hours)	Prior antiplatelet
Blend +	38.1 [18.2, 68.6]	1.9 [1.4, 3.0]	97 (26.5)
Blend -	10.9 [4.7, 25.4]	1.9 [1.4, 2.8]	493 (25.9)
P	<0.001	0.68	0.80
Black hole +	36.0 [19.6, 62.0]	1.8 [1.3, 2.6]	111 (26.8)
Black hole -	10.5 [4.6, 24.5]	2.0 [1.4, 3.0]	479 (25.8)
P	<0.001	0.003	0.67
Hypodensity +	32.1 [15.7, 59.6]	1.8 [1.3, 2.7]	185 (26.4)
Hypodensity -	8.9 [4.0, 19.2]	2.0 [1.4, 3.0]	407 (25.9)
P	<0.001	0.026	0.79
Island +	64.6 [39.3, 84.5]	2.0 [1.4, 3.1]	69 (34.5)
Island -	11.8 [5.00, 26.4]	1.9 [1.4, 2.8]	521 (25.1)
P	<0.001	0.21	0.004

*Data are median [interquartile range] or number (%). Analyses are Mann-Whitney U test and Chi-squared test. HV =haematoma volume

Supplemental Table III Multivariate logistic regression analysis for predictors of haematoma progression and total ICH expansion

Variables	*Adjusted OR (Model 1)	p	†Adjusted OR (Model 2)	p
**Haematoma progression**				
Blend sign	1.59 (1.20-2.11)	0.001	1.57 (1.19-2.08)	0.002
Black hole sign	1.56 (1.02-2.38)	0.039	-	-
Hypodensities	-	-	1.49 (1.07-2.07)	0.019
Island sign	0.94 (0.63-1.40)	0.76	0.95 (0.63-1.42)	0.79
**Total ICH expansion**				
Blend sign	1.64 (1.25-2.17)	<0.001	1.60 (1.21-2.12)	0.001
Black hole sign	1.97 (1.30-2.99)	0.001	-	-
Hypodensities	-	-	1.97 (1.41-2.76)	<0.001
Island sign	0.93 (0.63-1.37)	0.72	0.94 (0.64-1.38)	0.73

* Model 1 excludes hypodensities with adjustment for age, sex, premorbid mRS, systolic blood pressure, GCS, presence of IVH on baseline scan, lobar location, baseline haematoma volume, onset to CT time, prior antiplatelet therapy, treatment with tranexamic acid, black hole sign X haematoma volume. †Model 2 excludes black hole sign with adjustment of covariates similar to Model 1.
